# Construction of a one-step multiplex real-time PCR assay for the detection of serogroups A, B, and E of *Pasteurella multocida* associated with bovine pasteurellosis

**DOI:** 10.3389/fvets.2023.1193162

**Published:** 2023-06-28

**Authors:** Haojie Wang, Lingxiang Xin, Yang Wu, Yan Liu, Wensheng Yao, He Zhang, Yunhao Hu, Rendong Tong, Liangquan Zhu

**Affiliations:** ^1^China Institute of Veterinary Drug Control, Beijing, China; ^2^State Key Laboratory of Veterinary Biotechnology, Harbin Veterinary Research Institute of Chinese, Academy of Agricultural Sciences, Harbin, China

**Keywords:** bovine pasteurellosis, *Pasteurella multocida*, diagnosis, serogroup, one-step multiplex real-time PCR

## Abstract

Bovine pasteurellosis, caused by serogroups A, B, and E of *Pasteurella multocida* (Pm), is mainly manifested as bovine respiratory disease (BRD) and hemorrhagic septicemia (HS). The disease has caused a great economic loss for the cattle industry globally. Therefore, identifying the Pm serogroups is critical for optimal diagnosis and subsequent clinical treatment and even epidemiological studies. In this study, a one-step multiplex real-time PCR assay was established. Three pairs of specific primers were prepared to detect the highly conserved genomic regions of serogroups A (*HyaD*), B (*bcbD*), and E (*ecbJ*) of Pm, respectively. The results depicted that the method had no cross-reaction with other bovine pathogens (*Mannheimia hemolytica, Escherichia coli, Listeria monocytogenes, Staphylococcus aureus, Salmonella Dublin, Mycobacterium paratuberculosis*, infectious bovine rhinotracheitis virus, and *Mycoplasma bovis*). The linear range (10^7^ to 10^2^ copies/μL) showed the R^2^ values for serogroups A, B, and E of Pm as 0.9975, 0.9964, and 0.996, respectively. The multiplex real-time PCR efficiency was 90.30%, 90.72%, and 90.57% for CartA, CartB, and CartE, respectively. The sensitivity result showed that the serogroups A, B, and E of Pm could be detected to be as low as 10 copies/μL. The repeatability result clarified that an intra-assay and an inter-assay coefficient of variation of serogroups A, B, and E of Pm was < 2%. For the clinical samples, the detection rate was higher than the OIE-recommended ordinary PCR. Overall, the established one-step multiplex real-time PCR assay may be a valuable tool for the rapid and early detection of the serogroups A, B, and E of Pm with high specificity and sensitivity.

## 1. Introduction

*Pasteurella multocida* (Pm) is a Gram-negative bacterial pathogen in multi-host infections, including avian, cattle, and swine (1). In addition, Pm can be transmitted by mosquitoes, flies, or blood-sucking insects or by direct bites by cats, dogs, and other animals (2, 3). It has even been reported that people might be infected by ill animals in an unknown manner, highlighting its potential threat to human health (4–7). These diseases have caused huge economic losses to the cattle industry because of increased mortality, high treatment costs, reduced growth rate, and poor carcass quality (8). Pm can be classified into five serogroups (A, B, D, E, and F) based on the capsule antigen (K antigen) (9, 10). Isolates in different serogroups are associated with different diseases in different host species (11). For example, the majority of cases of fowl cholera are caused by serogroups A of Pm, atrophic rhinitis of pigs is associated predominantly with serogroups D isolates, bovine and porcine pneumonia are associated mainly with serogroups A, and hemorrhagic septicemia of cattle and water buffaloes is caused exclusively by serogroups B and E isolates (12, 13). Bovine pasteurellosis, caused by serogroups A, B, and E of Pm, is mainly manifested as bovine respiratory disease (BRD) and hemorrhagic septicemia (HS) (12). Reports of other serogroups (D and F) isolated from cattle are rare (14–16). In cattle, Pm infection usually results in BRD and HS. BRD is caused by serogroup A of Pm and is often co-infected with other pathogens such as *M. hemolytica* (17, 18). The course of BRD can range from 3 days to 1 week with respiratory symptoms such as dry cough, dyspnea, and fibrinous types of pneumonia (12). HS is caused by serogroups B and E of Pm (19, 20). HS shows high mortality for ill cattle with acute symptoms of high fever, diarrhea, and death within 12–24 h (12). According to the rapid deterioration of the disease, early diagnosis and serogroup identification are beneficial to managing and controlling Pm infection.

Currently, the diagnosis of Pm mainly includes cultural and biochemical methods, serotyping methods, and molecular biological methods. Although cultural and biochemical methods are regarded as the “gold standard” for diagnosis, it needs to collect samples from animals in the late stage of disease for bacterial culture, and it is not very easy to make a definitive diagnosis only via microscopic observation. In addition, the serotyping methods based on the indirect hemagglutination test, agar gel immunodiffusion, and ELISA are of great significance for the epidemiological investigation and the study of pathogenic regularity (9, 21, 22). However, this method is highly complicated and requires high serum concentration and titer requirements. With the development of molecular biology, gene amplification is one of the most reliable methods to identify Pm. The multiplex PCR method based on the Pm capsule-encoding gene cluster or lipopolysaccharide exokaryon-encoding gene cluster makes it possible to quickly define the serogroup of Pm in the clinic (23, 24). However, this method also has some drawbacks. The serogroup of some strains, which is defined as “untypable strain,” cannot be defined by this multiplex PCR assay (25–27). In addition, multilocus sequence typing (MLST) based on nucleic acid sequencing can quickly define the serogroup of target bacteria (28). Currently, two online databases are mainly used to define the Pm serogroup: multiple host MLST Databases and RIRDC MLST Databases (29, 30). Moreover, next-generation sequencing is recently used for the direct diagnosis of Pm (31). Although faster and more convenient, the serotyping methods MLST and DNA sequencing are not commonly used in the clinic due to their higher costs.

The real-time PCR assay uses specific probes to identify the target sequence with high accuracy. At the same time, the target sequence was double controlled by the primer and the probe, with good specificity and a low false positive ratio. Compared to the conventional PCR assay, the real-time PCR assay has higher sensitivity, faster speed, high throughput, and direct quantification. In particular, it has been reported that the development of an HS real-time PCR assay has the advantage of detecting multiple sequence types (ST) of Pm in a short time compared to the MLST method (20). Moreover, a multiplex real-time PCR assay was developed for gene amplification and detection in the same tube to avoid contamination (32, 33). Currently, there is no report on a one-step multiplex real-time PCR assay for detecting serogroups A, B, and E of Pm associated with the bovine pasteurellosis.

As reported by previous studies, Pm can be categorized into five serogroups (A, B, D, E, and F) based on the capsule antigen, and the genes related to capsule formation could serve as reliable markers for serotyping of Pm (34–36). It was found that, among all the genes related to capsular formation, *hyaD, bcbD*, and *ecbJ* proved to be specific targets to distinguish serogroups A, B, and E of Pm (23, 37). Therefore, we designed primers and probes using these three genes as specific targets to establish a multiplex real-time PCR method to simultaneously detect serogroups A, B, and E of Pm. The sensitivity, specificity, and repeatability of the method were proved, and the method was carried out for clinical application.

## 2. Materials and methods

### 2.1. Bacterial strains, viruses, and cultures

Pm was cultured in Martin broth medium at 37°C, while *Mannheimia. haemolytica* (*M. haemolytica*) was cultured in Martin broth supplemented with 5% fetal bovine serum at 37°C. Other bacterial strains were cultured in proper media under the recommended conditions. IBRV was propagated on Madin-Darby bovine kidney (MDBK) cells. The used bacterial strains are displayed in Table 1.

**Table 1:** Bacteria strains used in this study.

**Bacterial strains**	**Source**
*P. multocida* Cart A^*^	CVCC390 (bovine)
*P. multocida* Cart B^*^	CVCC391 (bovine)
*P. multocida* Cart E^*^	CVCC393 (bovine)
*M. haemolytica*	CVCC1655 (bovine)
*E. coli*	CVCC237 (bovine)
*L. Monocytogenes*	CVCC1597 (bovine)
*S. aureus*	CVCC3053 (bovine)
*S. enteriditis*	CVCC79351 (bovine)
*M. paratuberculosis*	CVCC1625 (bovine)
Infectious bovine rhinotracheitis virus, IBRV	CVCC AV1546 (bovine)
Mycoplasma bovis	Laboratory isolated (bovine)

CVCC, China Veterinary Culture Collection Center.

^*^Cart A, serogroup A of Pm; Cart B, serogroup B of Pm; Cart E, serogroup E of Pm.

### 2.2. Nucleic acid extraction

The total DNA of Pm, *M. haemolytica*, and other bacterial strains were extracted using the Genomic DNA TIANamp Bacteria DNA kit (TIANGEN, China). The total DNA of IBRV was extracted from cell cultures with the help of the Magnetic Viral DNA/RNA kit (TIANGEN, China). Finally, the extracted DNA was stored at −80°C for further use.

### 2.3. Primer and probe design

According to the specific regions of serogroups A, B, and E of Pm genomes (GeneBank, CP006976, LIUP01000027, AF302466), the multiple pairs of primers and probes were prepared using the Primer 5 software and verified for specificity by Primer-BLAST (Table 2). Primers and probes were synthesized by Sangon Biotech (Shanghai, China).

**Table 2:** Primers and probes of serogroups A, B, and E of Pm.

**Pathogens**	**Gene**	**Primers and probes**	**Sequences (5^′^end to 3^′^end)**	**Length**	**Accession no**.
Cart A	*HyaD*	Cart A-F	CAGTTTCTCTGGATTGGCGC	100 bp	CP006976
		Cart A-R	AAAGCAACATTACCCGCCG		
		Cart A-probe	FAM-CTCCGCTTATCCGATTCGCCTTTCC-BHQ1		
Cart B	*bcbD*	Cart B-F	GAAAATGCTATTCCTTTGACTGCTT	102 bp	LIUP01000027
		Cart B-R	AAGCTGATCACCTAATCCAAAACC		
		Cart B-probe	Cy5-AGCAGCACCTCCGTATTGACAGATACACC-BHQ2		
Cart E	*ecbJ*	Cart E-F	TCAGAAGACACTTTAGGAGAAAGAC	122 bp	AF302466
		Cart E-R	CAGAGCCATATCCGCTAAATAG		
		Cart E-probe	TAMRA-CAAGCAGCAAGCTATGCCAATGGT-BHQ2		

### 2.4. Construction of standard plasmids

The genome DNA of serogroups A, B, and E of Pm were used as the template to amplify the target fragments, and then, the amplicons were purified and cloned into the pMD18-T vector (TaKaRa, China). The primers used for amplification are described in Table 3. The constructed plasmids were subjected to positive transformation into *E. coli* DH5α competent cells (Vazyme Biotech, China). After being cultured for 18–20 h at 37°C, the plasmid was extracted utilizing the EndoFree Mini Plasmid Kit II (TIANGEN, China) and quantified using a NanoPhotometer^®^ (Thermo Fisher, USA). The plasmids were named pMD-CartA, pMD-CartB, and pMD-CartE and then stored at −80°C until use as standard plasmids. The copy numbers of plasmids were calculated with the help of the following formula:


$$\begin{array}{l} \text{Plasmid}\;\text{copies/}\mu \text{L = }(\text{6}.\text{02}\times {\text{10}}^{\text{23}})\times (X^{*}ng\text{/}\mu L\times {\text{10}}^{-\text{9}})\\ \text{ /constructed}\;\text{plasmid}\;\text{length}\;(\text{bp})\times \text{660}\; \end{array}$$

(32).

^*^X: Recombinant plasmid concentration

**Table 3:** Primers used for standard constructions.

**Pathogens**	**Gene**	**Primers**	**Sequences (5^′^end to 3^′^end)**	**Length**	**Accession no**.
Cart A	*HyaD*	F	ATGAATACATTATCACAAGCAATAAAAGC	2,918 bp	CP006976
		R	TTATAGAGTTATACTATTAATAATGAACTTGTTAACAG		
Cart B	*bcbD*	F	ATGCATAAAATTATTTTCTCACAAACAA	1,427 bp	LIUP01000027
		R	TCACTTGCCTACATTCATTTGGTT		
Cart E	*ecbJ*	F	ATGGACTCTAATGATAAATTAATTAAACTCAT	2,117 bp	AF302466
		R	TTATATAACAGTAATTTCTTCTGGGATTCC		

### 2.5. Optimization of the singleplex real-time PCR assay

The matrix method (Table 4) was adopted to optimize the singleplex real-time PCR assay parameters, such as the annealing temperature, the primer concentration, and the probe. The total volume of the PCR reaction is 20 μL, including 2 × Animal Detection U^+^ Probe qPCR Super Premix (Vazyme Biotech, China), 2 μL of positive plasmid template, and distilled water to a total volume of 20 μL. All reactions were amplified by a LightCycler^®^ 480 II instrument (Roche Life Sciences, Swiss), and the amplification parameters were: 37°C for 2 min; 95°C for 30 s; and then 40 cycles of 95°C for 10 s and annealing temperature and extension temperature (56°C, 57°C, 58°C, 59°C, and 60°C) for 30 s. The fluorescent signals were analyzed at the end of each cycle.

**Table 4:** Optimization of singleplex real-time PCR.

**TaqMan Probe (μmol/μL)**	**Forward and reverse Primers (**μ**mol/**μ**L)**				
	**2**	**4**	**6**	**8**	**10**
2	0.1/0.1^*^	0.1/0.2	0.1/0.3	0.1/0.4	0.1/0.5
4	0.2/0.1	0.2/0.2	0.2/0.3	0.2/0.4	0.2/0.5
6	0.3/0.1	0.3/0.2	0.3/0.3	0.3/0.4	0.3/0.5
8	0.4/0.1	0.4/0.2	0.4/0.3	0.4/0.4	0.4/0.5
10	0.5/0.1	0.5/0.2	0.5/0.3	0.5/0.4	0.5/0.5

^*^Final concentration of probe/forward and reverse (μmol/μL).

### 2.6. Optimization of the multiplex real-time PCR assay

The optimal reaction conditions of the multiplex real-time PCR, including primer and probe concentrations, were confirmed with the help of orthogonal experiments by referring to the optimal reaction conditions of the singleplex real-time PCR. Finally, the fluorescent signals were analyzed at the end of each cycle.

### 2.7. Standard curve analysis

Subsequently, 10-fold-diluted standard plasmids from 1 × 10^7^ copies/μL to 1 × 10^2^ copies/μL (3 replicates for each gradient) were selected for standard curve analysis. The amplification was constructed in the light of the optimized multiplex real-time PCR assay, and the standard curve was directly formed and derived from quantitative fluorescence software. The correlation coefficient (R^2^) and standard equation were calculated in the light of the amplification curve.

### 2.8. Specificity of the multiplex real-time PCR assay

The DNA of *M. haemolytica, E. coli, L. Monocytogenes, S. aureus, S. enteriditis, M. paratuberculosis*, IBRV, and *Mycoplasma bovis* were deemed as templates for the established multiplex real-time PCR to verify the specificity of the assay. In addition, the DNA of serogroups A, B, and E of Pm and distilled water were considered as the positive and negative controls, respectively.

### 2.9. Sensitivity of the multiplex real-time PCR assay

To assess the lowest detection limit, the standard plasmids of pMD-Cart A, pMD-Cart B, and pMD-Cart E were mixed, and then, the 10-time serial diluent from 1 × 10^7^ copies/μL to 1 × 10^0^ copies/μL was used as a template under the optimized conditions.

### 2.10. Repeatability of the multiplex real-time PCR assay

Three positive plasmids with different gradients (1 × 10^7^ copies/μL, 1 × 10^5^ copies/μL, and 1 × 10^3^ copies/μL) were equivalently mixed as templates and then detected by the established multiplex PCR. The intra-assay and inter-assay tests were all performed in triplicate, with an interval of 2 weeks. The intra-and inter-assay CVs were used as an index to evaluate the repeatability of the assay.

### 2.11. Detection of artificial simulation samples and clinical samples by the multiplex real-time PCR

Virulent strains CVCC390, CVCC391, and CVCC393 were cultured with Martin broth medium, then diluted to OD_600_≈0.6 with normal saline, and six mice were infected intraperitoneally with 1 × 10^4^ CFU bacteria (11). As a blank control, 3 mice were injected simultaneously with 0.3 mL Martin broth medium. After 48 h, the lung, liver, and spleen were collected from the six dead mice. Approximately 10-fold sterilized PBS (0.01 mol Ligue pH 7.4, containing 1 mmol/L LEDTA) was added to tissue homogenate. The TIAamp Genomic DNA kit (TIANGEN, China) extracted the genome for multiplex real-time PCR detection.

Approximately 147 nasal swab samples were collected from the asymptomatic cattle, and 59 nasal swab samples were collected from the cattle with signs of a fever (41–42°C), respiratory distress, depression, red and swollen eyes, and lacrimation, in Xinjiang, China, from December 2021 to January 2022. Total DNA was extracted according to the Hi-Swab DNA kit (TIANGEN, China) and subsequently tested for the presence of serogroups A, B, and E of Pm using the established multiplex real-time PCR. In addition, the above DNA samples were also tested by regular PCR, as recommended for Pm identification (Chapter 3.4.10) in the OIE Manual (38).

## 3. Results

### 3.1. Optimal reaction conditions for the multiplex real-time PCR assay

After optimization using the matrix method, the fluorescence intensity and the CT value of each possible combination were compared. The optimized singleplex real-time PCR reaction conditions are shown in Table 5, with the optimal annealing temperature determined to be 59°C. The optimal reaction system and procedure were obtained by testing the primer concentration, probe concentration, and annealing temperature based on the optimized singleplex real-time PCR reaction conditions. The developed assay utilized a 20 μL reaction mixture, consisting of 10 μL of 2 × Animal Detection U^+^ Probe qPCR Super Premix (Vazyme Biotech, China), 2.0 μL of total DNA (DNA of three pathogens), together with the optimum primer and probe concentration for three serotypes shown in Table 6, and distilled water to a total volume of 20 μL. The amplification parameters were 37°C for 2 min, 95°C for 30 s, and then 40 cycles of 95°C for 10 s and 59°C (annealing temperature and extension temperature) for 30 s.

**Table 5:** Optimum singleplex real-time PCR reaction conditions.

**Pathogens**	**Final concentration (**μ**mol/**μ**L)**	
	**Forward and reverse primer**	**TaqMan probe**
Cart A	0.5	0.3
Cart B	0.6	0.3
Cart E	0.6	0.6

**Table 6:** Optimum primer and probe concentrations.

**Pathogens**	**Final concentration (**μ**mol/**μ**L)**	
	**Forward and reverse primer**	**TaqMan probe**
Cart A	0.4	0.3
Cart B	0.4	0.5
Cart E	0.5	0.3

### 3.2. Construction of the standard curve

**Figure 1:**
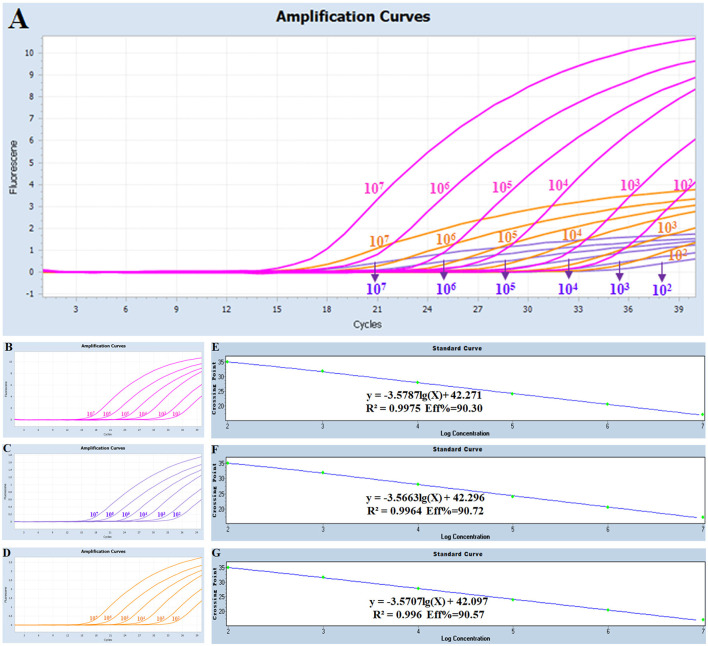
Amplification and standard curves for the multiplex real-time PCR: **(A)** amplification curves (X-axis: Cycle, Y-axis: Fluorescence) for the multiplex real-time PCR; **(B–D)** amplification curves (X-axis: Cycle, Y-axis: Fluorescence) of Cart A, Cart B, and Cart E for each positive plasmid standard with concentrations ranging from 1 × 10^7^ copies/μL to 1 × 10^2^ copies/μL; **(E–G)** standard curves of positive plasmid standards of Cart A, Cart B and Cart E. **(E)** Cart A: Y = -3.5787 lg(X)+42.271 R^2^ = 0.9975 Eff% = 90.30; **(F)** Cart B: Y = -3.5663 lg(X)+42.296 R^2^ = 0.9964 Eff% = 90.72; **(G)** Cart E: Y = -3.5707 lg(X)+42.097 R^2^ = 0.996 Eff% = 90.57.

Three standard plasmids with dilution from 1 × 10^7^ copies/μL to 1 × 10^2^ copies/μL (three replicates for each gradient) were mixed in equal volume and then amplified under the optimized multiplex real-time PCR condition. The corresponding slope of the equation, correlation coefficient (R^2^), and amplification efficiency (E) were −3.5787, 0.9975, and 90.30% for serogroups A of Pm, respectively; −3.5663, 0.9964, and 90.72% for serogroups B of Pm, respectively; and −3.5707, 0.996, and 90.57% for serogroups E of Pm, respectively (Figure 1). Collectively, an excellent linear relationship (R^2^ ≥ 0.990) was confirmed between the initial template concentrations and the corresponding threshold cycle (Ct) values.

### 3.3. Specificity of the multiplex real-time PCR assay

**Figure 2:**
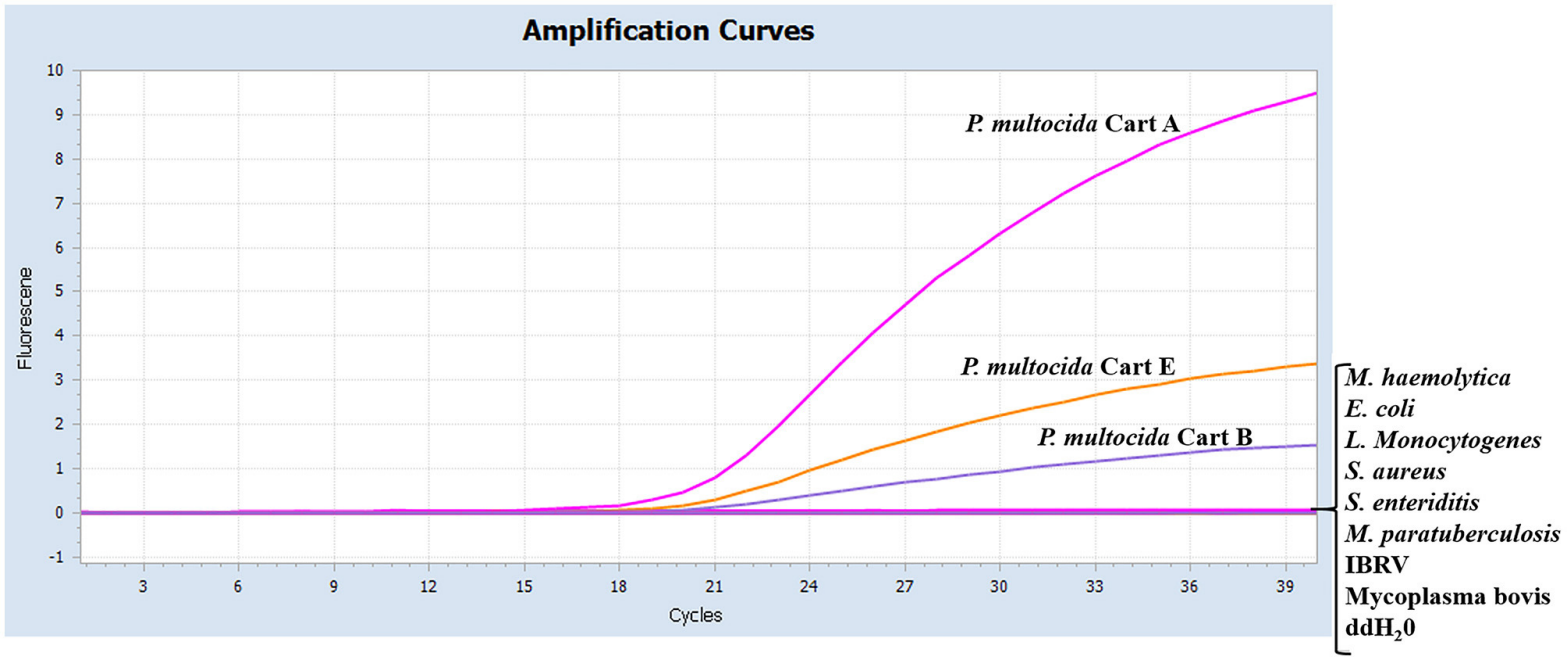
Specificity of the real-time multiplex PCR using different pathogens. The data were represented with amplification curves (X-axis: Cycle, Y-axis: Fluorescence). The serotypes A, B, and E of Pm*, M. haemolytica, E. coli, L. Monocytogenes, S. aureus, S. enteriditis, M. paratuberculosis*, IBRV, and Mycoplasma bovis were detected by the real-time multiplex PCR.

As shown in Figure 2, serogroups A, B, and E of Pm had specific amplification curves, while the other eight pathogens and the negative control had no amplification or fluorescent signal. It was indicated that the multiplex real-time PCR assay was specific for serotyping of Pm.

### 3.4. Sensitivity and repeatability of the multiplex real-time PCR assay

**Figure 3:**
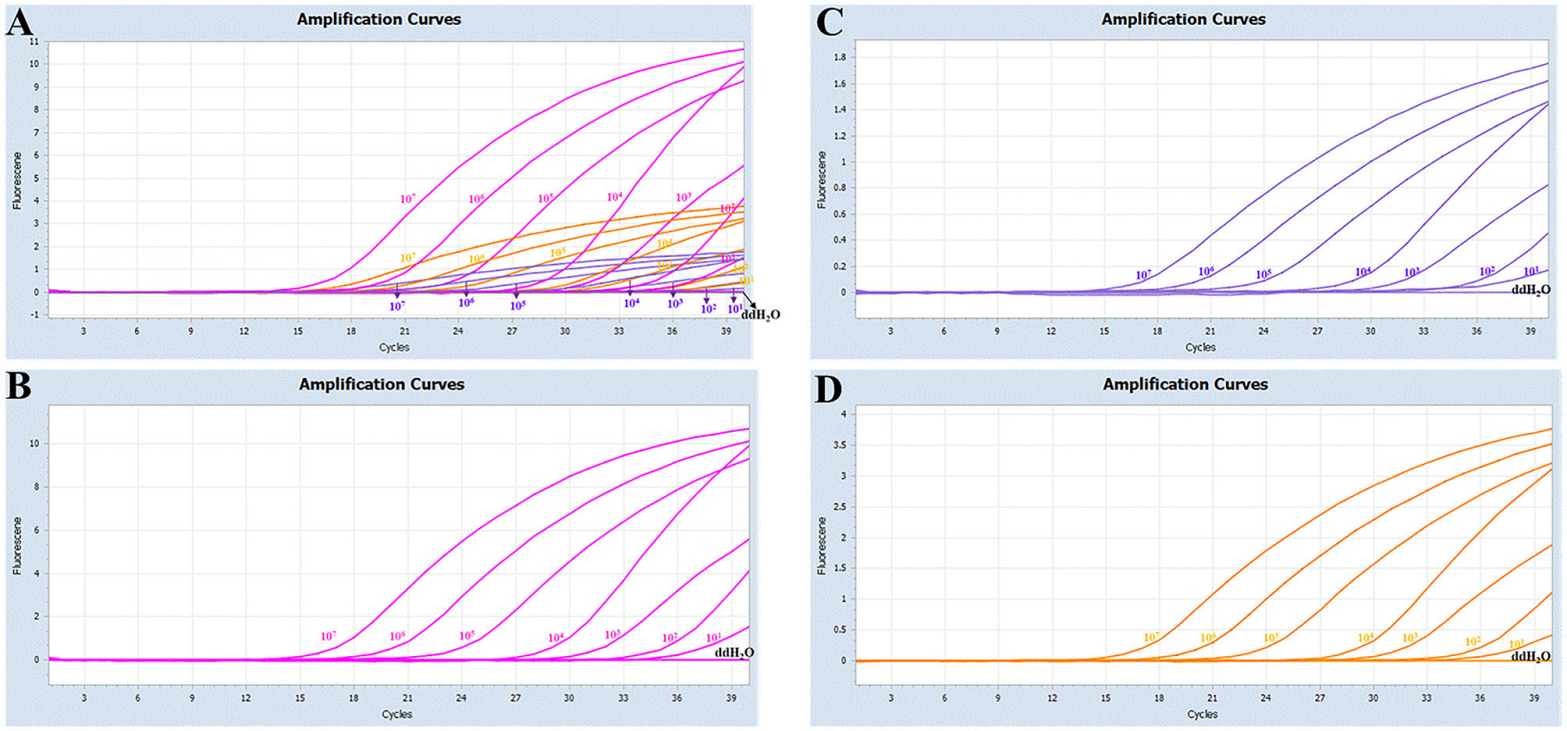
**(A)** Sensitivity of the multiplex real-time PCR assay; **(B–D)** amplification curves (X-axis: Cycle, Y-axis: Fluorescence) of Cart A, Cart B, and Cart E for each positive plasmid standard with concentrations ranging from 1 × 10^7^ copies/μL to 1 × 10^1^ copies/μL.

The sensitivity studies described that the detection limit of this method for Pm Cart A, Pm Cart B, and Pm Cart E was 10 copies/μL (Figure 3). The cutoff Ct value for Pm CartA and CartE was 37, indicating that the sample with a Ct value less than or equal to 37 ( ≤ 37) was considered positive but the sample with a Ct value higher than 37 (>37) was considered negative. The cutoff Ct value for Pm CartB positivity was 38 (Figures 3A–C). As shown in Table 7, the coefficient of variation was < 2%, describing that the assay had good repeatability.

**Table 7:** Repeatability of the multiplex real-time PCR assay.

**Standard plasmid**	**Concentration of template (copies/**μ**L)**		**Intra-coefficient of variation**		**Inter-coefficient of variation**
		**X** ±**SD**	**CV (%)**	**X** ±**SD**	**CV (%)**
pMD-Cart A	10^7^	17.250 ± 0.061	0.35	17.547 ± 0.040	0.23
	10^5^	23.437 ± 0.100	0.43	23.510 ± 0.286	1.21
	10^3^	31.810 ± 0.060	0.19	31.337 ± 0.202	0.65
pMD-Cart B	10^7^	17.370 ± 0.262	1.51	17.530 ± 0.040	0.23
	10^5^	24.230 ± 0.270	1.11	24.787 ± 0.176	0.71
	10^3^	31.080 ± 0.036	0.12	31.677 ± 0.176	0.12
pMD-Cart E	10^7^	17.660 ± 0.191	1.08	17.477 ± 0.153	0.87
	10^5^	23.277 ± 0.230	0.99	23.467 ± 0.233	0.99
	10^3^	31.570 ± 0.303	0.96	31.580 ± 0.384	1.22

### 3.5. Detection of artificial simulation samples and clinical samples by the multiplex real-time PCR assay

For the six artificial simulation samples, the accuracy of the detection method was 100% (Table 8). To further verify its clinical applicability for the serotyping of Pm, 59 nasal swabs collected from the cattle with symptoms of fever and respiratory distress and 147 nasal swabs collected from the asymptomatic cattle were tested (Table 8). Among the total 206 clinical samples, 60 (29.13%) were detected as positive for type A, and eight (3.88%) were detected as positive for type B. It corresponds with the epidemiological of BRD and HS in China as that serogroup A is still predominantly prevalent, and serogroup E is still not present in cattle in China (14, 39). In detail, the nasal swabs collected from the tattles with disease symptoms had the same detection rate (serogroup A: 21/59; serogroup B: 5/59) for the multiplex real-time PCR and ordinary PCR (Table 8). However, in the detection of nasal swabs collected from asymptomatic cattle, the detection rate of the multiplex real-time PCR (serogroup A: 39/147; serogroup B: 3/147) was higher than that of the ordinary PCR (serogroup A: 31/147; serogroup B: 2/147). Thus, the establishment of a diagnostic method with higher sensitivity is essential for the timely prevention and control of the spread of the disease.

**Table 8:** Test results of clinical samples and artificial simulation samples.

**Samples**	**The multiplex real-time PCR**		Reference methods^*^	**Agreements**
	**Serotype**	**Positive**	**Positive**	
18 samples of an artificial simulated infection	A	6/6 (100%)	6/6 (100%)	100%
	B	6/6 (100%)	6/6 (100%)	
	E	6/6 (100%)	6/6 (100%)	
3 negative control samples	A, B, and E	0	0	
59 nasal swab samples (symptomatic)	A	21/59 (35.59%)	21/59 (35.59%)	100%
	B	5/59 (8.47%)	5/59 (8.47%)	100%
	E	0/59 (0%)	0/59 (0%)	100%
147 nasal swabs samples (asymptomatic)	A	39/147 (26.53%)	31/147 (21.08%)	79.48%
	B	3/147 (2.04%)	2/147 (1.36%)	66.66%
	E	0/147 (0%)	0/147 (0%)	100%
	negative	138/206 (66.99%)	147/206 (71.36%)	93.88%

^*^The reference methods refer to the ordinary PCR that was recommended for Pm (Chapter 3.4.10) identification by the OIE (OIE Terrestrial Manual 2021).

## 4. Discussion

We developed a one-step multiplex real-time PCR assay to detect serogroups A, B, and E of Pm using *HyaD, bcbD*, and *ecbJ* as marks, respectively. This method exhibited high specificity, sensitivity, and reliability, with no cross-reactions observed with other bovine pathogens and a detection limit of 10 copies/μL. The coefficient of variation was also determined to be < 2%. Moreover, this method effectively differentiated Pm infections of different serogroups (A, B, and E) in symptomatic and asymptomatic animals, facilitating early clinical diagnosis of bovine pasteurellosis and enabling rapid molecular epidemiological investigation.

The capsule is not only one of the main components on the surface of Pm but also one of its important virulence factors, which plays a crucial role in the anti-phagocytosis of Pm (40). The whole genome sequence analysis of Pm has identified capsular-specific genes for different serogroups: *hyaD* for serogroup A, *bcbD* for serogroup B, *dcbF* for serogroup D, *ecbJ* for serogroup E, and *fcbD* for serogroup F (37, 41). The capsular genotyping method was developed based on PCR detection of cap genes, using primers highly specific for different serogroups, and genotypes determined by this multiplex capsular PCR-based typing system were reliably and accurately distinguished among the podoconjugate serogroups (23).

Petersen et al. developed a real-time PCR assay that specifically detects HS-associated isolates based on the gene of EST, capable of detecting serotypes B: 2 and E: 2 (20). Moustafa et al. confirmed the high sensitivity and specificity of HS-LAMP detection in HS-associated Pm by utilizing the established PM-LAMP and HS-LAMP assays for serogroup B:2 strains associated with HS in cattle and buffalo (42). Liu et al. developed a duplex real-time PCR assay for assessing Pm, which exhibited high specificity and sensitivity with a detection limit of 10 copies/μL. However, this method can only identify Pm and capsular serogroup A (43), lacking the ability to simultaneously detect serogroups A, B, and E of Pm in infected cattle.

The serogroups A:1 and A:3 caused BRD in cattle under other bacteria or virus co-infection, weaning pressure, poor management, diet change, sudden weather change, transportation and auction, and other contributing factors (44). HS is caused by serotype B:2 in Asia and serotype E:2 in Africa, with disease outbreaks mostly occurring under monsoon conditions in Asian countries. Currently, the serotype E of Pm is prevalent mainly in African countries, such as Egypt (16). In addition, the isolation of serogroup E from diseased cattle has been reported in other countries, such as England and Wales (45). Serogroup A of Pm has the highest prevalence among cattle herds in China, followed by serogroup B of Pm. However, serogroup E of Pm has not been found in cattle herds in China (14, 39). The serogroup A of Pm in clinical samples still dominates the epidemiological trend in cattle (Table 8), which is consistent with the current epidemiological situation in China. However, with the need for international trade and quarantine introduction, it is risky for the serogroup E of Pm to be introduced into China. The one-step multiplex real-time PCR assay showed a higher detection rate compared to the ordinary PCR assay, especially for the clinical samples collected from the asymptomatic cattle (Table 8). With the higher sensitivity, the multiplex real-time PCR assay was capable of detecting low Pm concentrations, with a detection limit of 10 copies/μL (Figure 3). It is reasonable that the asymptomatic cattle, carrying lower bacterial concentrations at preclinical stages, are amenable to detection through the multiplex real-time PCR assay but not through ordinary PCR techniques. Compared to other samples (blood, heart, liver, and so on), swabs are the most easily collected and monitored samples for risk assessment and epidemiological investigation of the disease. However, the effectiveness of nucleic extraction varies due to the differences in swab quality, methods for collection, preservation, and nucleic acid extraction. Moreover, the ordinary PCR assays demonstrated lower sensitivity than the one-step multiplex real-time PCR assay, leading to decreased detection rate.

Bovine pasteurellosis is mainly prevented and controlled through vaccination, stress reduction, timely diagnosis, and disinfection. Given the acute symptoms and rapid deterioration of the disease, rapid and early diagnosis of Pm serogroups during the latent period and the early stage of the infection might improve chances for clinical therapy and disease control. Therefore, rapid typing and identification of Pm in the clinic will be beneficial to understand its infection and pathogenic regularity and realize pasteurellosis monitoring, cross-regional correlation analysis, the molecular epidemiology, and traceability of infectious diseases.

## 5. Conclusion

This study developed an efficient multiplex real-time PCR assay for the simultaneous detection of serogroups A, B, and E of Pm causing bovine pasteurellosis, which provided technical support for clinical detection, epidemic prevention examination, epidemiological investigation, and vaccine evaluation.

## Data availability statement

The original contributions presented in the study are included in the article/supplementary material, further inquiries can be directed to the corresponding author.

## Ethics statement

The animal study was reviewed and approved by Animal Ethics Committee of the China Institute of Veterinary Drug Control.

## Author contributions

LZ, YL, and WY conceived and designed the experiments. HW, LX, YH, and RT performed the experiments, analyzed the data, and prepared figures and tables. HZ and YW provided clinical samples. All authors reviewed drafts of the paper and approved the final draft.
